# Supplementary material for: Evolutionary and Expression Analysis of MOV10 and MOV10L1 Reveals Their Origin, Duplication and Divergence
Source: Int J Mol Sci. 2022 Jul 7;23(14):7523. doi: 10.3390/ijms23147523 (PMC9319325; doi:10.3390/ijms23147523)
Supplement: Supplementary file 1 [file ijms-23-07523-s001.zip › Figure S1.pdf]

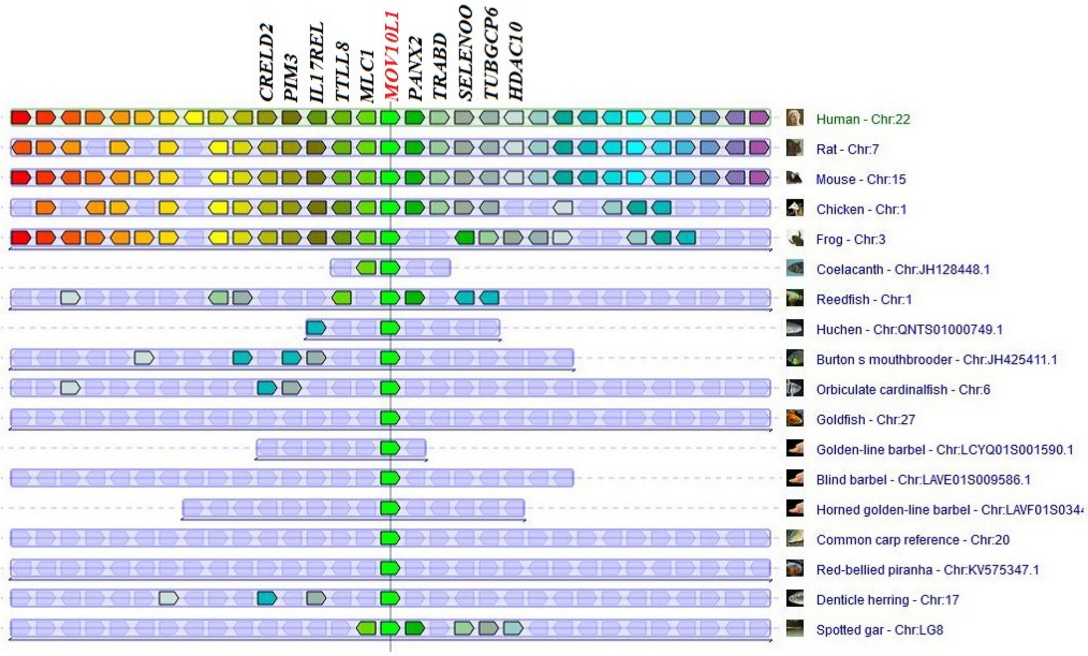

Figure S1: The synteny analyses of *MOV10L1* genes among human, rat, mouse, chicken frog and some fishes. The bright, multi-colored squares represent the homologous genes between different species. Those faint pale blue squares represent non-homologous genes. The direction of the square represents the direction of the gene. The Figure was collected and download from Genomicus v100.01 (https://www.genomicus.bio.ens.psl.eu/genomicus-100.01/cgi-bin/search.pl, accessed on 27 December 2021).
